# Dental wastewater reveals a hidden reservoir of oral bacteriophage diversity

**DOI:** 10.1128/spectrum.01820-26

**Published:** 2026-08-12

**Authors:** Carli Roush, Marvin Whiteley

**Affiliations:** 1 Center for Microbial Dynamics and Infection, Georgia Institute of Technology 1372, Atlanta, Georgia, USA; 2 School of Biological Sciences, Georgia Institute of Technology 1372, Atlanta, Georgia, USA; 3 Emory-Children's Cystic Fibrosis Center 200748, Atlanta, Georgia, USA; University of Florida College of Dentistry, Gainesville, Florida, USA

**Keywords:** phageome, oral microbiome, bacteriophage, viral metagenomics, periodontal disease, dental wastewater

## Abstract

Bacteriophages (phages) are being explored as alternatives or complements to antibiotics because of their ability to selectively kill bacterial pathogens. However, phages that infect many oral bacteria remain undiscovered. Here, we discovered that dental wastewater harbors previously underexplored phage diversity. Viral particles concentrated from dental wastewater displayed diverse morphologies, including abundant filamentous phage-like particles. Deep long-read metagenomic sequencing of concentrated viral particles generated 7.4 billion bases of sequence data and yielded 255 medium- to high-quality viral operational taxonomic units (vOTUs), including 46 predicted complete genomes. Comparison with large phage databases revealed that 63 of these 255 vOTUs had no detectable match, indicating that extensive sequencing of dental wastewater substantially expands the number of potential bacteriophages associated with the human oral microbiome. Host prediction linked many vOTUs to oral-associated bacterial taxa, including species with few or no previously reported phages, such as *Porphyromonas gingivalis*, *Tannerella forsythia*, and *Candidatus* Saccharibacteria. Functional annotation identified diverse genes associated with antiphage defense systems within a subset of vOTUs, suggesting that oral phages may contribute to the movement of genes encoding bacterial immune functions within the oral microbiome. Together, these findings expand the known oral phageome and show that dental wastewater contains a largely untapped diversity of phages.

**IMPORTANCE:**

The human oral cavity contains a diverse microbial community, but the bacteriophages (phages) that infect many oral bacteria remain poorly characterized. This gap limits our understanding of how phages shape oral microbial communities. Here, we show that dental wastewater is an underexplored source of oral phage diversity. Deep long-read metagenomic sequencing revealed 255 medium- to high-quality phage operational taxonomic units, many of which are not present in existing oral phage databases. These genomes include predicted phages of periodontal disease-associated bacteria and other oral taxa with few or no known phages. Dental wastewater therefore expands the known human oral phageome and reveals candidate phages linked to bacteria associated with oral health and disease.

## INTRODUCTION

Bacteriophages (phages) are viruses that infect bacterial cells (1). Rising antibiotic resistance and the need for more selective antimicrobials have renewed interest in phages as therapeutic agents (2–4). Historically, phage discovery has relied heavily on enrichment and isolation using culturable bacterial hosts (5). Although this approach has been successful, it captures only a fraction of viral diversity because phage isolation depends on both the availability of a culturable bacterial host and permissive host–phage interactions under laboratory conditions. Many phages have narrow host ranges, sometimes infecting only specific strains within a bacterial species (6), and many bacteria remain difficult to culture under standard laboratory conditions, limiting access to the phages that infect them (7). These challenges have motivated a shift toward culture-independent approaches, with metagenomic sequencing becoming an important approach for detecting phages without first isolating their bacterial hosts (8).

Phages that infect many human oral bacterial species remain difficult to isolate, and numerous oral-associated taxa have no known phages (9–12), making the oral microbiome a strong candidate for culture-independent discovery of new phages. Previous metagenomic studies have used saliva, dental plaque, and oral mucosal samples to identify novel oral phages (13, 14). Here, we examined dental wastewater, the fluid generated during dental cleaning procedures, as an underexplored source for discovering novel phages associated with the oral microbiome. Dental wastewater differs from saliva, plaque, or mucosal swabs in that it can be collected in large volumes as a naturally pooled sample of oral-derived material generated during routine dental procedures.

Using viral particle enrichment and long-read metagenomic sequencing, we characterized phage diversity in dental wastewater. From 7.4 billion bases of long-read sequencing data, we identified 255 medium- or high-quality viral operational taxonomic units (vOTUs), including 46 vOTUs predicted to be complete (15). Comparison with large phage databases showed that while a subset of dental wastewater vOTUs overlapped with previously described oral phages, many were absent from the database, and no vOTUs were represented at a species level in any database analyzed (16). We also annotated genes associated with antiphage defense systems within the vOTUs, highlighting oral phages as potential vehicles for the movement of bacterial immune functions within the oral microbiome (17, 18). Together, these findings expand the number of phages associated with the human oral microbiome and demonstrate the value of dental wastewater for uncovering previously underrecognized oral phage diversity.

## RESULTS

### Transmission electron microscopy reveals morphologically diverse phage-like particles in dental wastewater

**Fig 1:**
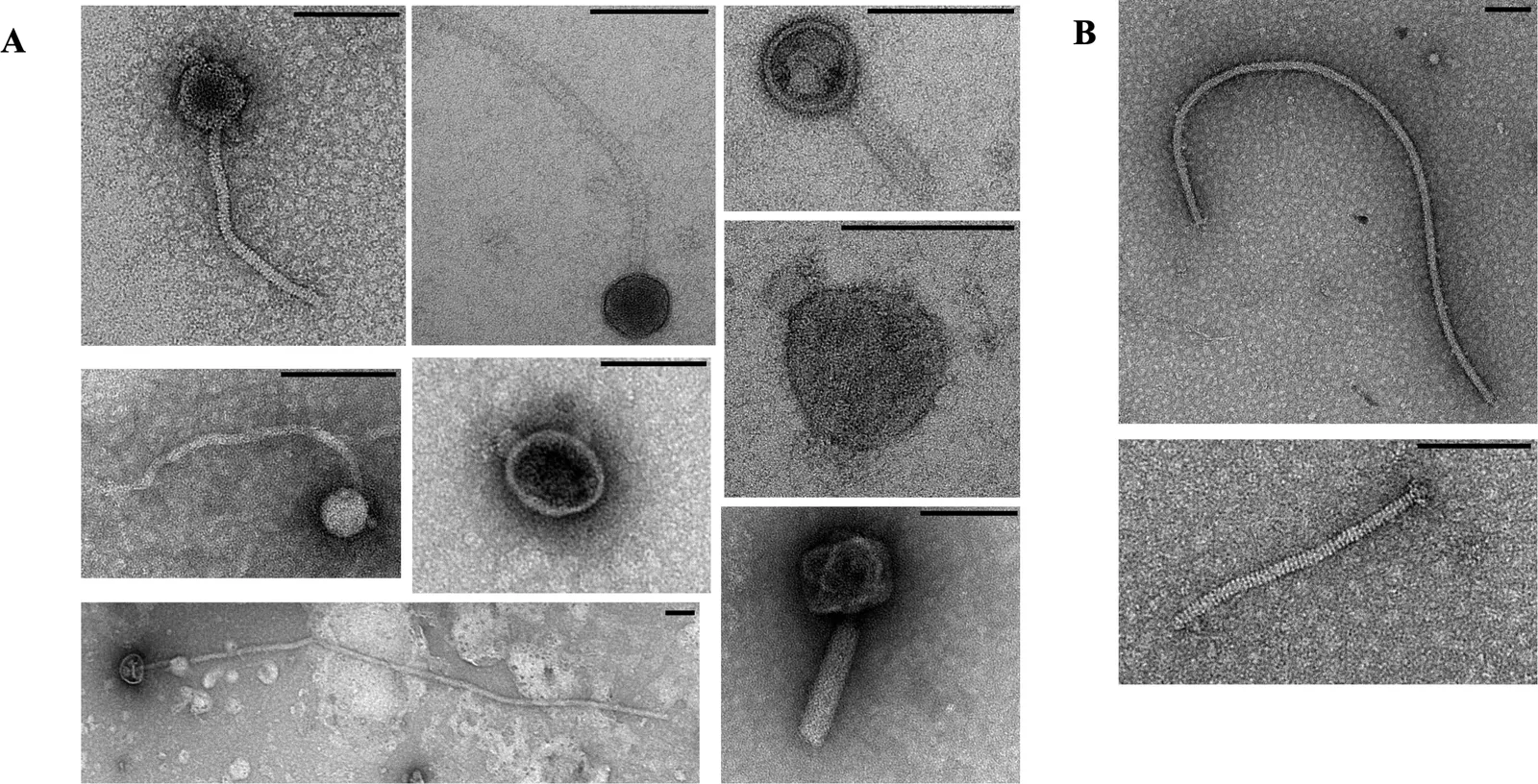
Transmission electron microscopy reveals morphologically diverse phage-like particles in dental wastewater. Representative transmission electron micrographs of viral particle preparations from dental wastewater. Panels show morphologically diverse phage-like particles, including particles with head–tail morphologies (**A**) and filamentous structures (**B**). Filamentous phage-like particles were observed in multiple fields of view and varied substantially in length. Black scale bars in the upper right corner of each panel represent 100 nm.

Dental wastewater was obtained from a mobile dental practice operated by Senior Smiles GA directly after dental cleaning procedures at a senior assisted living facility in Alpharetta, GA, USA. Dental wastewater represents a pooled environmental sample generated from routine dental clinic procedures, including saliva, oral debris, irrigation fluids, and material collected through dental suction systems during patient care. Approximately 400 mL of wastewater was collected after treatment with chlorhexidine by Senior Smiles GA (concentration unknown). The sample was stored at 4°C for several weeks before processing (19). After low-speed centrifugation and filtration to remove large particles and intact bacterial cells, phages were concentrated using ultracentrifugation. Transmission electron microscopy (TEM) of this concentrated material revealed phage-like particles with diverse morphologies in dental wastewater (Fig. 1A). Filamentous phage-like particles were especially abundant and ranged from approximately 200 nm to 4 µm in length (Fig. 1B). Concentration of phage particles in the resuspended ultracentrifuged dental wastewater was estimated using polystyrene beads as a known concentration reference. Through this method, we estimate the total phage concentration to be approximately 5.58 × 10⁹ particles/mL ± 7.1 × 10⁸ particles/mL. Filamentous phage particles were approximately twice as abundant as Caudoviricetes-like phage particles (3.68 × 10⁹ particles/mL ± 5.1 × 10⁸ particles/mL and 1.91 × 10⁹ particles/mL ± 3.2 × 10⁸ particles/mL, respectively).

### Long-read viral metagenomics identifies 255 medium- to high-quality vOTUs

**Fig 2:**
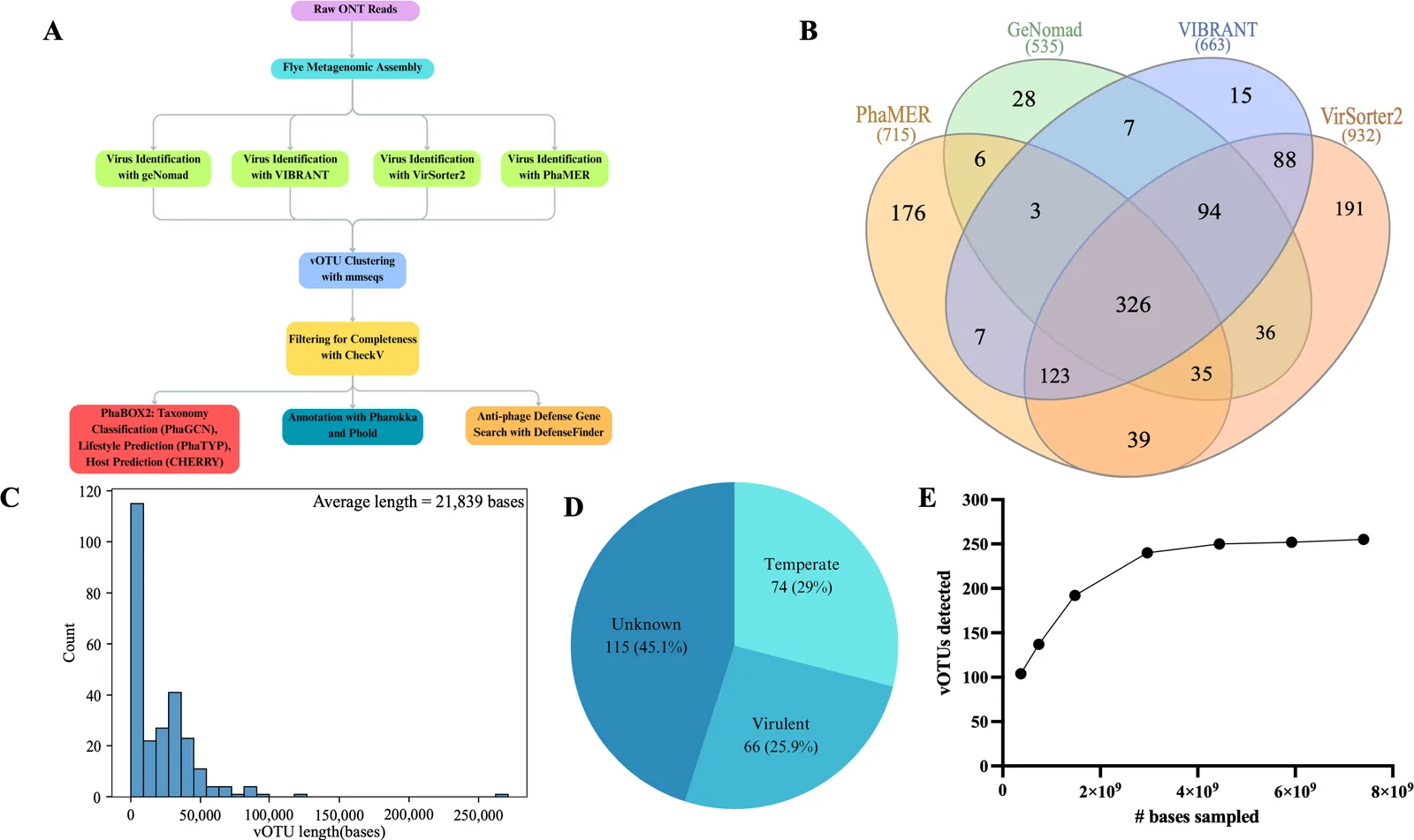
Long-read viral metagenomic workflow and summary of dental wastewater viral operational taxonomic units (vOTUs). (**A**) Computational workflow used to identify and analyze vOTUs from dental wastewater sequencing data. (**B**) Overlap among vOTUs identified by the four viral prediction tools (GeNomad, VIBRANT, VirSorter2, and PhaMER). (**C**) Length distribution of representative sequences for the 255 medium- to high-quality vOTUs retained after CheckV filtering. The average representative sequence length was 21,839 bp. (**D**) Predicted lifestyle distribution of the 255 vOTUs. (**E**) Sequencing saturation curve showing the number of vOTUs detected as a function of total bases sequenced. Points correspond to subsamples containing 5%, 10%, 20%, 40%, 60%, 80%, and 100% of total metagenomic reads obtained in this study.

We generated 7.4 billion total bases of sequence data from the concentrated dental wastewater using long-read sequencing, which enabled assembly of multiple phage genomes. Figure 2A outlines the computational workflow used to assemble and annotate the viral genomes from dental wastewater. Long-read assemblies generated with Flye were analyzed with four viral identification tools: PhaMER, GeNomad, VIBRANT, and VirSorter2 (20–23). Because viruses lack universal marker genes and much of their sequence diversity remains uncharacterized, detection from metagenomic data is less standardized than bacterial genome identification (24, 25). In addition, viruses also tend to have higher mutation rates than bacteria, and the majority of viral diversity remains uncharacterized, commonly referred to as “viral dark matter” (26). We therefore used these four prediction tools, which produced distinct but overlapping sets of contigs (Fig. 2B) (27). Predicted phage contigs were clustered into vOTUs using thresholds of ≥95% average nucleotide identity and ≥85% alignment, and the longest contig from each cluster was selected as the representative sequence. After filtering for medium- or high-quality prokaryotic viral assemblies using CheckV, 255 vOTUs remained. The contig retention rate after filtering for completeness for each tool is PhaMER: 139/715 (19.4%) contigs retained, geNomad: 164/535 (30.7%) contigs retained, VIBRANT: 160/663 (24.1%) contigs retained, and VirSorter 2: 234/932 (25.1%). While VirSorter2 identified the most retained vOTUs, it also had the highest number of discarded contigs. GeNomad had the highest retention rate of the four tools.

The 255 vOTUs varied in length, with an average genome size of 21,839 bp (Fig. 2C). Viral lifestyle predictions were generated using PhaTYP, predicting that 74/255 (29%) of vOTUs are temperate; 66/255 (25.9%) are virulent; and the remaining vOTUs (115/255 [45.1%]) are unknown (Fig. 2D) (28). Host prediction with CHERRY assigned putative hosts to 117 vOTUs (29), whereas 138 vOTUs had no predicted bacterial host (Table 1). Among vOTUs with predicted hosts, many were assigned to bacterial genera commonly found in the oral cavity, with *Streptococcus* and *Prevotella* (30–32) representing the most frequent predicted host genera. Finally, sequencing saturation analysis indicated that additional sequencing would likely recover relatively few additional vOTUs using our detection criteria (Fig. 2E) (33).

**TABLE 1:** Predicted bacterial hosts of vOTUs

Predicted host taxonomy	Count
Unknown	138
*Streptococcus*	12
*Prevotella*	11
*Campylobacter*	10
*Actinomyces*, *Candidatus* Saccharibacteria	6 each
*Veillonella*, *Pseudomonas*, *Lactobacillus*	5 each
*Actinobaculum*, *Treponema*	4 each
*Rothia, Desulfobulbus, Pseudopropionibacterium*	3 each
*Selenomonas*, *Tannerella*, *Eubacterium*, *Polaribacter*, *Ralstonia*, *Neisseria*	2 each
*Clostridioides*, *Xylanimonas*, *Bordetella*, *Kiritimatiella*, *Acinetobacter*, Bacteroidetes bacterium oral taxon 272; *Blastochloris*, *Porphyromonas*, *Megasphaera*, *Gilliamella*, *Capnocytophaga*, *Mesorhizobium*, *Propionibacterium, Mogibacterium*, *Maribacter*, uncharacterized bacterium YEK0313, *Faecalibacterium*, *Arthrobacter*, *Winogradskyella*, *Abiotrophia*, *Atopobium*, *Vreelandella*, *Fretibacterium*, *Bifidobacterium*, unclassified bacterium endosymbiont of *Bathymodiolus*, *Corynebacterium*, *Fusobacterium*, *Centipeda*	1 each

### Unique vOTUs are present in dental wastewater

**Fig 3:**
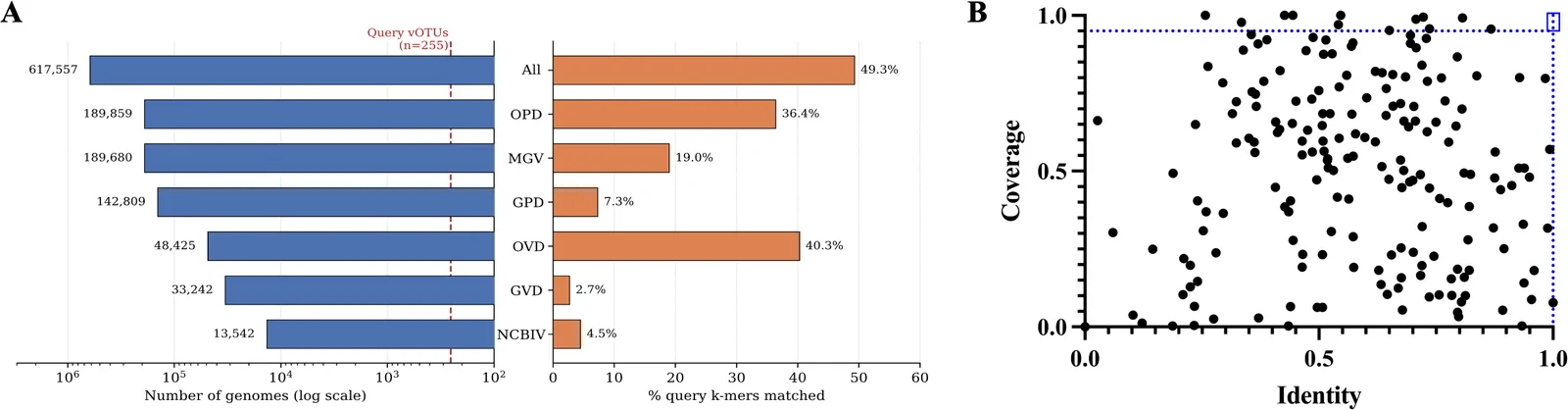
Dental wastewater vOTUs are largely distinct from sequences in existing databases. Dental wastewater vOTUs were compared to the Oral Phage Database (OPD), Oral Virus Database (OVD), Gut Phage Database (GPD), Gut Virome Database (GVD), NCBI Virus “Bacteriophage” Database (NCBIV), and Metagenomic Gut Virus Database (MGV). (**A**) Schematic comparing the number of sequences in each database and the percentage of query k-mers that had matches in each database, as well as all six databases combined (All) with the 255 dental wastewater vOTUs. (**B**) Dot plot showing the coverage and identity of the single best alignment from the combined databases against each vOTU. Sixty-three vOTUs had no detectable alignment (position 0, 0). The blue box in the upper right corner denotes graph space, which would represent a species-level alignment (full coverage and 0.95 or greater identity).

To determine whether dental wastewater captured phage diversity not represented in existing oral metagenomic data sets, we compared the 255 dental wastewater vOTUs to the Oral Phage Database (OPD), Oral Virus Database (OVD), Gut Phage Database (GPD), Gut Virome Database (GVD), NCBI Virus Database, and Metagenomic Gut Virus Database (MGV) with Sourmash (16, 34–39). We also combined all six databases and compared them against the dental wastewater vOTUs. The OPD and OVD had the highest overlaps (36.4% and 40.3% of query k-mers matched to each database, respectively), while the GVD had the lowest overlap (2.7% of query k-mers matched to database) (Fig. 3A). When the databases were combined, 49.3% of the total dental wastewater vOTU k-mers overlapped with known sequences (Fig. 3A). Minimap2 analysis against the combined databases showed that 63 of the 255 dental wastewater vOTUs (24.7%) we identified had no detectable match in the combined database. After mapping to the combined databases with minimap2, we extracted the single best alignment for each vOTU (maximized query coverage and identity scores). None of the 255 vOTUs were fully covered (100% query coverage) at ≥95% nucleotide identity by any single viral genome in the combined database, suggesting that these vOTUs are not represented in current reference collections at the species level (Fig. 3B). Thus, although dental wastewater vOTUs partially overlap with previously described phages, approximately one-quarter of them had no detectable overlap with phage genomes in the six large viral databases, and none of the vOTUs were represented at a species level. These findings indicate that dental wastewater captures phage diversity that has been previously missed by gut-, saliva-, and oral tissue-based metagenomic surveys.

### Dental wastewater vOTUs contain genes that encode antiphage defense proteins

**Fig 4:**
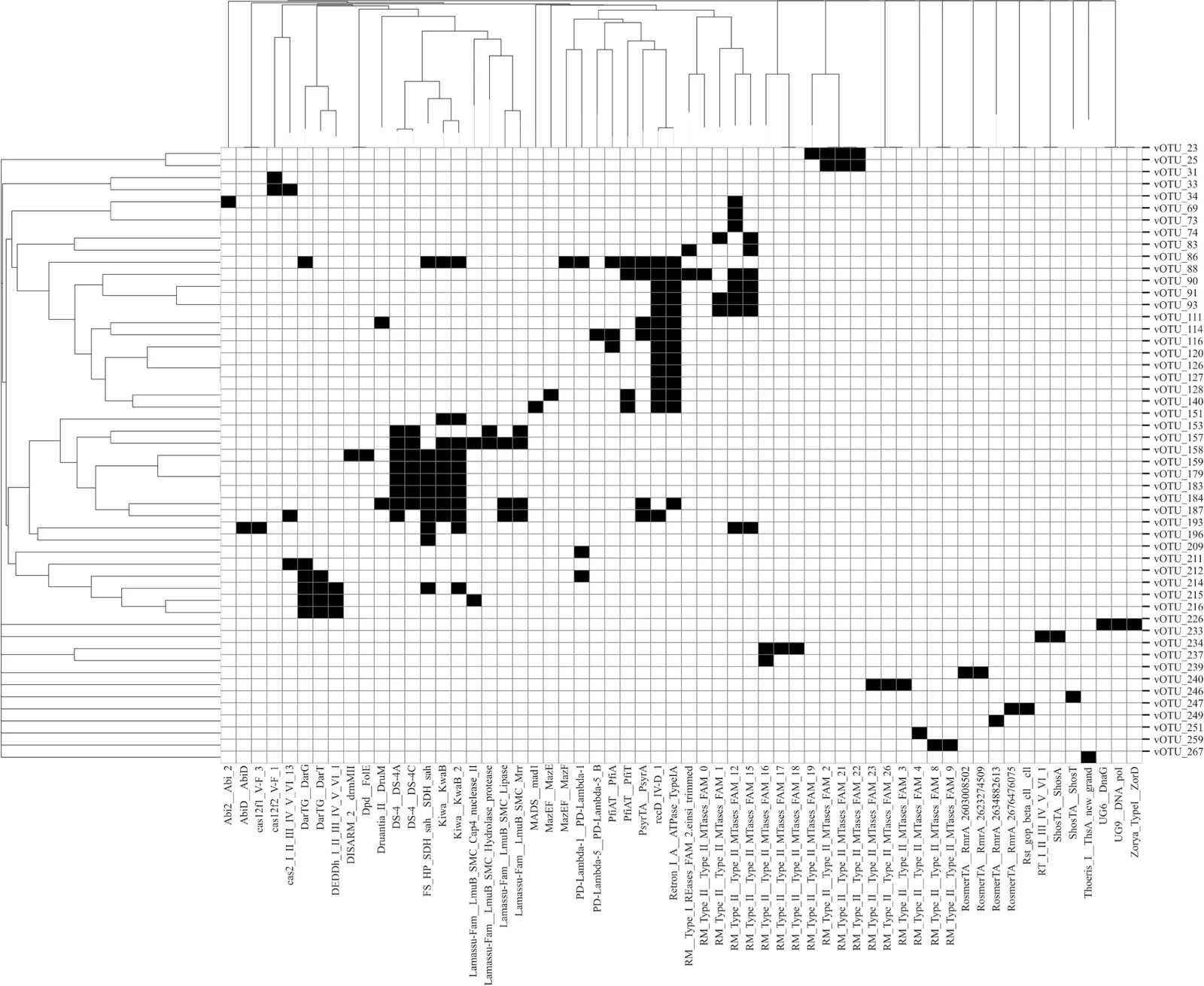
Dental wastewater vOTUs contain genes associated with antiphage defense systems**.** Presence/absence matrix of antiphage defense-associated genes identified in representative vOTU sequences using DefenseFinder. Rows represent vOTUs containing at least one defense-associated gene, and columns represent detected defense-associated genes. Black squares indicate the presence of a given gene in a vOTU, and white squares indicate absence. vOTUs and genes were clustered by Jaccard distance to visualize patterns of co-occurrence among defense-associated genes.

Because phage-mediated lateral DNA transfer plays an important role in transferring phage defense genes between bacterial hosts (18), we next asked whether dental wastewater vOTUs carried genes associated with antiphage defense. Using DefenseFinder (40), we identified 60 unique defense-related genes distributed across 51 of the 255 vOTUs. These genes represented several major classes of bacterial immune systems, including restriction-modification, abortive infection, CRISPR-Cas, and toxin–antitoxin (40). To visualize patterns of co-occurrence, defense-associated genes and vOTUs were clustered in a presence/absence matrix using Jaccard distance (Fig. 4) (41, 42). This analysis revealed that the DEDDh_I_II_III_IV_V_VI_1 gene, which encodes 3′−5′ exonuclease, commonly co-occurs with the DarTG system toxin–antitoxin system (43, 44). In addition, two genes belonging to a predicted but experimentally unconfirmed defense system (DS-4_DS-4A and DS-4_DS-4C) consistently co-occur with FS_HP_SDH_sah_SDH_sah, Kiwa_KwaB, and Kiwa_KwaB_2 (40, 45). Furthermore, we found that vOTUs containing recD_IV-D_1, involved in bacterial adaptive immunity, often co-occur with Retron_I_A_ATPase_TypeIA, which is part of a retron defense system (46–48). Together, these results indicate that a subset of dental wastewater vOTUs carry diverse defense-associated genes, including combinations of defense systems that co-occur within individual vOTUs.

### Genome annotation and host prediction of vOTUs in dental wastewater

To examine individual viral genomes in greater detail, we selected 25 vOTUs for annotation and phylogenetic analysis based on contig length, CheckV-estimated completeness, and sequence novelty (15). Complete information for the selected vOTU is provided in Table 2 and all vOTUs in Table S1. vOTUs included predicted phages of bacterial species for which phages have been predicted but not isolated, as well as species for which no phage sequences have been reported.

**TABLE 2:** Genomic features, predicted hosts, and closest viral relatives of selected vOTUs recovered from dental wastewater

	Genome length (bp)	Predicted host	Taxonomy	AAI completeness (%)	Closest viral BLAST hit accession	Closest viral query cover (%)	Closest viral percent identity (%)	Predicted lifestyle
vOTU_110	32,071	*Candidatus* Saccharibacteria	Caudoviricetes	100	BK030310	59	93.95	Virulent
vOTU_111	121,458	*Veillonella atypica*	Caudoviricetes	58.6	BK034824	84	95.83	Virulent
vOTU_114	271,192	Unknown	Caudoviricetes	100	NA^*a*^	NA	NA	Virulent
vOTU_116	63,355	*Tannerella forsythia*	Caudoviricetes	10	BK020970	0	77.73	Virulent
vOTU_120	60,751	*Prevotella buccae*	Caudoviricetes	97.6	BK039769	8	76.84	Virulent
vOTU_121	36,432	Unknown	Caudoviricetes	96.3	BK058954	86	91.54	Temperate
vOTU_127	51,945	*Kiritimatiella glycovorans*	Caudoviricetes	94.2	BK050848	7	87.6	Virulent
vOTU_133	34,454	*Eubacterium infirmum*	Caudoviricetes	100	BK046139	89	93.45	Temperate
vOTU_150	34,632	Unknown	Caudoviricetes	99.3	BK040341	97	92.38	Temperate
vOTU_161	37,627	Unknown	Caudoviricetes	100	BK058336	66	94	Temperate
vOTU_179	33,459	*Candidatus* Saccharibacteria	Caudoviricetes	94.6	BK029710	98	91.63	Virulent
vOTU_180	35,143	Unknown	Caudoviricetes	94.3	BK042180	59	91.4	Virulent
vOTU_187	30,495	*Centipeda felix*	Caudoviricetes	95.1	BK039679	59	96.66	Virulent
vOTU_191	25,158	*Porphyromonas gingivalis*	Caudoviricetes	67.8	BK038565	82	87.39	Temperate
vOTU_228	38,493	*Rothia dentocariosa*	Caudoviricetes	100	BK034376	94	97.67	Virulent
vOTU_235	94,037	Unknown	Caudoviricetes	100	BK034691	91	93.56	Virulent
vOTU_239	36,690	*Treponema socranskii*	Caudoviricetes	100	BK037056	63	90.37	Temperate
vOTU_240	49,827	*Candidatus* Saccharibacteria	Caudoviricetes	100	BK041650	83	96.69	Temperate
vOTU_257	36,099	*Streptococcus parasanguinis*	Caudoviricetes	92.5	BK038136	86	98.04	Temperate
vOTU_267	54,788	*Desulfobulbus oralis*	Caudoviricetes	86.8	OP030929	0	82.18	Temperate
vOTU_36	33,605	Unknown	Caudoviricetes	92	BK033790	41	94.95	Temperate
vOTU_74	86,827	*Pseudomonas aeruginosa*	*Citexvirus*	100	OR222680	78	87.93	Temperate
vOTU_82	38,826	*Candidatus* Saccharibacteria	Caudoviricetes	98.8	BK040969	54	90.82	Temperate
vOTU_83	74,917	*Prevotella baroniae*	Caudoviricetes	71.4	BK050874	15	74.86	Temperate
vOTU_92	32,640	*Candidatus* Saccharibacteria	Caudoviricetes	94	BK035786	36	95.11	Temperate

^*a*^ NA, not significant.

**Fig 5:**
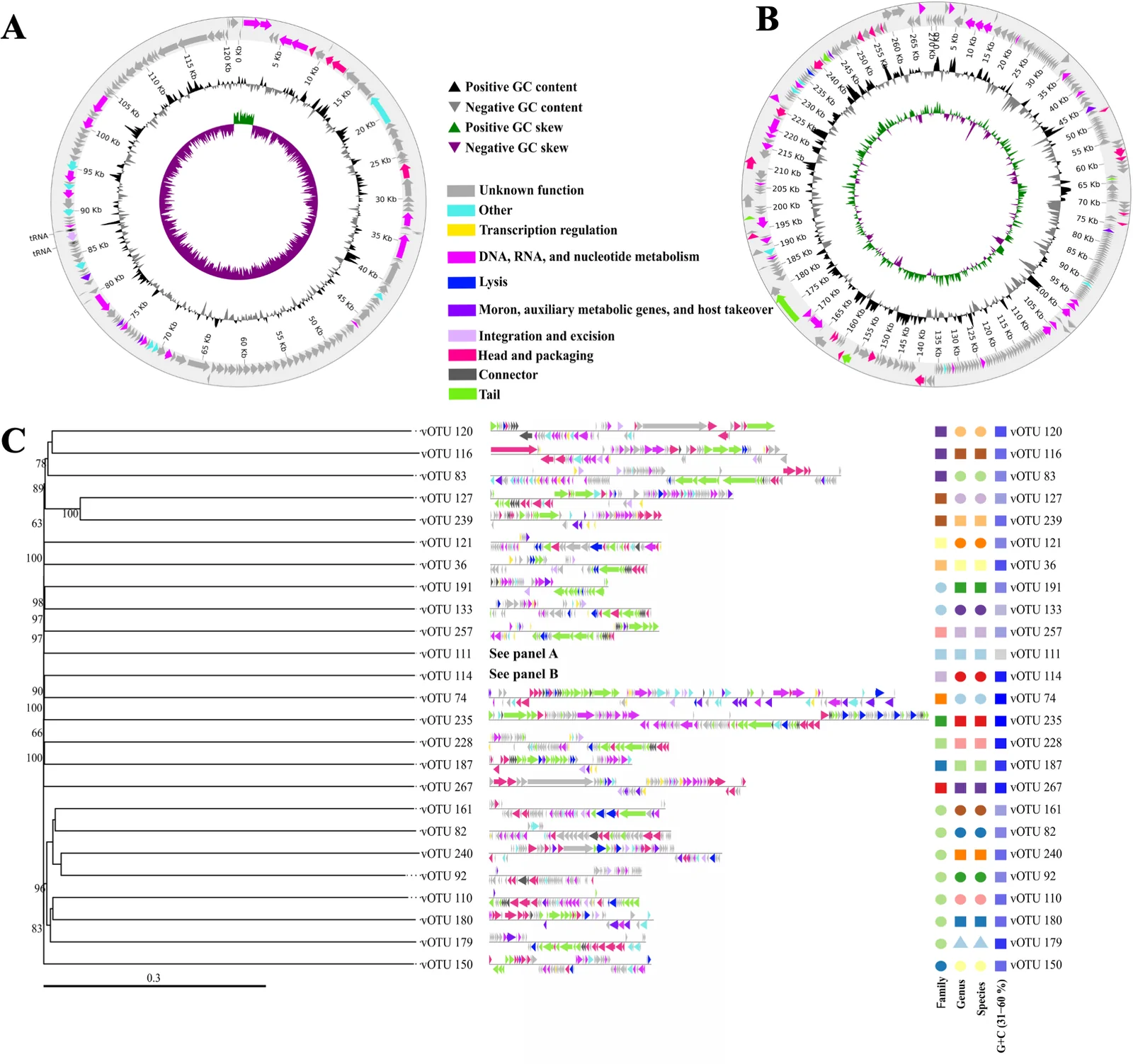
Genome annotation and phylogenetic relationships of selected dental wastewater vOTUs. Twenty-five vOTUs were selected for detailed annotation and phylogenetic analysis based on genome length, CheckV-estimated completeness, and sequence novelty. (**A and B**) Circular genome maps for two large selected vOTUs. Predicted coding sequences are colored by functional category. GC content and GC skew are shown as inner tracks. (**C**) VICTOR-based phylogenetic analysis of the selected vOTUs with linear genome annotation maps displayed alongside the tree. Colored arrows indicate predicted coding sequences and their functional categories.

Genome annotations for the 25 selected vOTUs were generated with Pharokka and Phold and visualized by functional category (Fig. 5A through C). Consistent with their broad sequence diversity, the selected vOTUs were generally distantly related to one another in VICTOR-based phylogenetic analysis (Fig. 5C). VICTOR assigned some vOTUs to the same viral family, including vOTU_161, vOTU_82, vOTU_240, vOTU_92, vOTU_110, vOTU_180, and vOTU_179; vOTU_191 and vOTU_133; vOTU_127 and vOTU_239; and vOTU_120, vOTU_116, and vOTU_83. However, no two selected vOTUs were assigned to the same genus or species. Based on BLAST comparisons to the closest viral matches, each of the 25 selected vOTUs fell below the 95% whole-genome nucleotide identity threshold commonly used for viral species demarcation, supporting their classification as novel viral species (Table 2). Because of their large genome sizes, vOTU_111 and vOTU_114 are shown in separate panels (Fig. 5A and B). vOTU_111 encoded 159 predicted coding DNA sequences (CDSs), 127 of which were annotated as hypothetical proteins, whereas vOTU_114 encoded 308 predicted CDSs, including 212 hypothetical proteins.

Several selected vOTUs were linked to oral taxa for which phages remain poorly characterized. Five vOTUs, vOTU_110, vOTU_179, vOTU_240, vOTU_82, and vOTU_92, were predicted to infect *Candidatus* Saccharibacteria (formerly known as TM7 taxon), a lineage of ultrasmall episymbiotic bacteria common in the human oral cavity. vOTU_111 was predicted to infect *Veillonella atypica* (49), representing the first predicted virulent phage sequence associated with this species (50). Several selected vOTUs did not receive a host prediction from CHERRY, although BLAST analysis suggested similarity between vOTU_180 and *Candidatus* Saccharibacteria (51). vOTU_114 was especially notable because it represents a jumbo phage with no significant similarity to sequences in the BLAST nr database (51).

Other selected vOTUs were predicted to infect oral bacteria associated with periodontal disease or dental infections. vOTU_116 was predicted to infect *Tannerella forsythia*, a subgingival species associated with periodontal tissue destruction (52, 53). vOTU_120 and vOTU_83 were predicted to infect *Prevotella buccae* and *Prevotella baroniae*, respectively, both of which are associated with periodontal or dental infections and lack isolated phages (31). vOTU_133 was predicted to infect *Eubacterium infirmum* (54), an asaccharolytic species isolated from periodontal pockets, and vOTU_187 was predicted to infect *Centipeda felix*, a species associated with deep periodontal pockets (52).

Several additional vOTUs were linked to oral bacteria with limited phage representation. vOTU_119 was predicted to infect *Porphyromonas gingivalis*, a periodontal pathogen for which only one active temperate phage has been isolated (55). vOTU_228 was predicted to infect *Rothia dentocariosa*, an oral species with only one previously characterized infective phage (56, 57). vOTU_239 was predicted to infect *Treponema socranskii*, which is associated with aggressive periodontitis and has predicted but no isolated phages (11, 58). vOTU_257 was predicted to infect *Streptococcus parasanguinis*, an early dental colonizer with no known or predicted phages (59, 60), and vOTU_267 was predicted to infect *Desulfobulbus oralis*, a sulfate-reducing periodontal pathobiont (61). Notably, vOTU_267 showed only limited similarity to its closest viral BLAST match, with 82.18% identity across less than 1% of the genome, highlighting its novelty.

Although most selected vOTUs were linked to oral-associated bacteria, the data set also included predicted phages of non-oral or broadly distributed taxa. For example, vOTU_74 was predicted to infect *Pseudomonas aeruginosa* and represents a putative novel species within the genus *Citexvirus*, indicating that dental wastewater can also recover phages associated with bacteria not specific to the oral cavity.

## DISCUSSION

This study shows that deep sequencing of dental wastewater substantially expands the catalog of bacteriophages associated with the human oral microbiome. From 7.4 billion bases of long-read sequence data, we recovered 255 medium- to high-quality vOTUs, including 46 predicted complete genomes. By purifying and sequencing genetic material from the viral particles themselves, the amount of “viral noise” from deprecated prophage regions within bacteria in the sample was limited. Comparison with six large databases built from thousands of human oral and gut metagenomes showed that many dental wastewater vOTUs were not detected in existing oral phage data sets, indicating that oral phage diversity remains incompletely sampled despite substantial sequencing efforts. Our discovery of new vOTUs in dental wastewater is likely due to both the depth of sequencing performed here and the nature of dental wastewater itself, which can be collected in large volumes and contains oral material dislodged during dental cleaning, including material from biofilm-associated, subgingival, and low-abundance communities that may be underrepresented in conventional oral samples. Thus, expanding the range of oral sample types studied, together with deep sequencing, may be critical for discovering new oral phages. Given the great diversity in oral microbiomes across humans, we expect that analysis of other geographically and temporally disparate dental wastewater samples would lead to further novel phage discovery.

The detection of defense-associated genes in a subset of dental wastewater vOTUs provides further support for the observation that oral phages may shape bacterial physiology beyond host lysis (18, 62). Antiphage defense systems are often viewed as bacterial barriers to phage infection, but phages and other mobile genetic elements can also mobilize defense-related genes between bacterial hosts. Clustering defense genes by Jaccard similarity revealed several co-occurring patterns, including associations between DEDDh-family exonuclease genes and DarTG toxin–antitoxin systems (43, 44), as well as co-occurrence of DS-4-associated genes with Kiwa-related defense genes (40, 45). These co-occurrence patterns provide a foundation for future studies aimed at determining whether defense-associated genes encoded by oral phages are functional, mobile, and capable of influencing phage biology.

Although filamentous phage-like particles were not a primary focus of this study, transmission electron microscopy revealed that they were highly abundant in dental wastewater (Fig. S1). Filamentous phages can influence bacterial physiology, biofilm formation, virulence, and horizontal gene transfer (63, 64), even though they are generally not candidates for conventional phage therapy (65). Well-studied examples include Pf phages in *Pseudomonas aeruginosa* biofilms (66, 67) and CTXϕ in *Vibrio cholerae* (68). Filamentous phages have also been adapted for phage display, nanotechnology, and biomaterial applications. Thus, while our data do not define the identity, hosts, or functions of the filamentous phages observed here, their apparent abundance suggests that dental wastewater may be a useful source for future studies focused on filamentous phage biology.

An important limitation of this study is that the analysis was performed on a single dental wastewater sample and that bacterial hosts of the vOTUs were computationally predicted rather than experimentally confirmed. Therefore, these data do not define the full diversity, prevalence, or geographic distribution of phages present in dental wastewater, nor do the host predictions establish infectivity or host range. Instead, the goal of this study was to determine whether deep sequencing of dental wastewater could reveal previously unrecognized phage diversity. Because dental wastewater represents a composite sample collected from the clinical environment, environmental microorganisms and their associated phages may also contribute to the observed viral community. The recovery of 255 medium- to high-quality vOTUs, 63 of which had no detectable matches in large phage databases, suggests that dental wastewater is a useful source for oral phage discovery. Although we hypothesize that phage novelty in dental wastewater is a repeatable result, we cannot be certain with one sample. Additionally, the lack of replicates precluded formal statistical analysis. Further, although filamentous phages were approximately twice as abundant as head–tail phages, only 3 of the 255 vOTUs were identified as belonging to the Inoviridae (filamentous) family. Therefore, this methodology may miss genomic diversity of filamentous phages within samples. Future studies that include broader sampling across dental practices, patient populations, and geographic locations, together with targeted isolation and host-range testing, will be needed to define the activity and host associations of these candidate phages.

## MATERIALS AND METHODS

### Preparation of dental wastewater

Approximately 140 mL from the ~400 mL total sample of dental wastewater was centrifuged at 10,000 × *g* for 10 min to sediment large particles. The resulting supernatant was then syringe-filtered through a 0.22 µm filter to remove remaining bacteria. Using the Beckman Optima XPN-100 Ultracentrifuge, the filtered supernatant was ultracentrifuged at 100,000 × *g* for 1 h at room temperature. Then, the supernatant was removed from the resulting pellet, which was resuspended in 3 mL phage buffer (10 mM Tris, 10 mM MgSO_4_, and 68 mM NaCl). Resuspension was achieved by soaking the pellet in phage buffer overnight at 4°C, then at room temperature for 2 h while shaking (200 rpm). Resuspended viral particles were stored at 4°C.

### Virus imaging

Five microliters of concentrated dental wastewater was pipetted onto a carbon grid coated in formvar and carbon with a mesh size of 300 (VWR #100491-116). After 60 s, the sample was wicked off using filter paper. Then, 5 µL of 2% uranyl acetate solution was pipetted onto the grid and wicked off after 30 s. Grids were allowed to dry completely. Transmission electron microscopy was carried out on a Hitachi HT7700 operating at 120 kV.

### Virus quantification

Polystyrene latex bead stock (Sigma LB5, 0.47 µm mean diameter, 10% solid content, density 1.05 g/mL) was used as an internal standard to determine phage concentration by TEM. Bead stock concentration was calculated from the manufacturer’s solid content and particle size specifications. Bead volume was calculated assuming a spherical particle of diameter 0.47 µm (radius = 0.235 µm = 2.35 × 10^−^⁵ cm): *V* = (4/3)πr³ = 5.44 × 10^−^¹⁴ cm³. Bead mass was calculated using the manufacturer-specified density (1.05 g/cm³): *m* = *V* × *ρ* = 5.71 × 10^−^¹⁴ g. Bead stock concentration was then calculated from the solid content (10% wt/vol = 0.1 g/mL): stock concentration = 0.1 g/mL ÷ 5.71 × 10^−^¹⁴ g = 1.75 × 10¹² beads/mL. The bead stock was diluted 1:500 in nuclease-free water to yield a working bead concentration of 3.5 × 10⁹ beads/mL. Resuspension of ultracentrifuged dental wastewater was mixed 1:1 with working bead stock and stained and imaged as described above. Beads and phage particles were counted in 100 fields of view at 30,000× magnification. Phage concentration was calculated using the following formula:


$$Conc\_phage=(\frac{N\_phage}{N\_bead})\times Conc\_bead,working$$

This calculation assumes phage and beads were mixed at equal volume ratios, such that the dilution introduced during mixing affects both stocks equally and cancels out. Counting uncertainty was estimated using Poisson statistics, as particle counts from electron micrographs are assumed to follow a Poisson distribution. For each phage type, the relative standard deviation (SD) of the phage-to-bead ratio was calculated as


$$Relative\;SD=\sqrt{\frac{1}{N\_phage}+\frac{1}{N\_bead}}$$

This relative SD was applied to the calculated concentration to obtain the absolute SD.

### Viral DNA extraction and sequencing

To remove non-viral DNA and RNA, 450 µL of resuspended viral particles was combined with 1 µL DNase I and 50 µL of DNase I 10× buffer (Promega, M6101), as well as 1 µL RNase A (10 mg/mL; Thermo Scientific, EN0531), then incubated at 37°C without shaking for 1.5 h. Twenty microliters of 0.5 M EDTA (final concentration 20 mM) was added to inactivate the nucleases. To digest the phage capsids, 1.25 µL of Proteinase K (20 mg/mL; Thermo Scientific, EO0491) was added, then the sample was incubated at 56°C for 1.5 h without shaking. In a fume hood, 520 µL of the bottom layer of phenol:chloroform:isoamyl alcohol reagent (25:24:1; Thermo Scientific, 1559303) was added to the sample before shaking vigorously by hand for at least 20 s. Then, the sample was centrifuged at room temperature for 5 min at 16,000 × *g* before 450 µL of the aqueous (upper) phase was transferred to a fresh microcentrifuge tube. Fifty microliters of 3 M sodium acetate (0.3 M final concentration) and 1,000 µL of 100% ethanol were added, and the sample was stored overnight at −20°C to precipitate DNA. After overnight precipitation, the sample was centrifuged at 17,200 × *g* at 4°C for 30 min, then the supernatant was carefully removed. The pellet was washed twice by adding 750 µL of 70% ethanol and centrifuging at 17,200 × *g* at 4°C for 2 min. After washing, the supernatant was carefully removed; residual ethanol was allowed to evaporate; and the DNA pellet was dissolved in nuclease-free water.

Because the concentration of the extracted DNA was too low to directly sequence, multiple displacement amplification–whole-genome amplification (MDA-WGA) was carried out using the EquiPhi29 DNA Amplification kit (Thermo Scientific, A65393). Five separate rounds of MDA-WGA were done following the manufacturer’s protocol, with 1 µg of input DNA in each round. It is important to note that MDA-WGA can introduce amplification bias, so no claims of individual vOTU abundance within the dental wastewater sample can be made. All five MDA-WGA amplifications were sent to Plasmidsaurus for sequencing using the bacterial whole-genome service. Amplification-free long-read sequencing libraries were constructed using v14 Nanopore library prep chemistry, and then primer-free sequencing was carried out using R10.4.1 flow cells. Base calling was done using Dorado (v4.3) Super-Accurate Basecalling with default Q10 quality filtering.

### Metagenomic analysis of viral sequences

Raw ONT sequencing reads (fastq.gz) were downloaded from Plasmidsaurus, and all five files were concatenated into one combined file. Total sequencing depth was 7.4 billion bases. Default parameters were used for each tool, except where specified otherwise. All tools were run on the Georgia Institute of Technology Partnership for an Advanced Computing Environment (PACE) by default. The Galaxy web instance of Flye (v2.9.6) was used to assemble the combined reads with –raw and –meta parameters selected (69, 70). Four separate tools for virus identification were used. VIBRANT (v1.2.1) and GeNomad (v1.11.1) were run on Galaxy, while VirSorter2 (v2.2.4) and PhaMER (v2.1.13) were run using PACE (20–23). Predicted viral contigs were clustered into vOTUs using MMseqs2 (v13.45,111) with cutoffs of ≥95% average nucleotide identity and ≥85% alignment (27, 71). The longest contig in each vOTU was chosen to be the representative sequence. Then, CheckV (v1.0.3) was used to assess genome completeness (15). Only genomes predicted to be 50% complete or greater were used for further analysis. Taxonomy classification (PhaGCN), lifestyle prediction (PhaTYP), and host prediction (CHERRY) were carried out on the PHABOX2 web server (v2.1.13) (22, 28, 72, 73). Twelve eukaryotic virus sequences were manually removed, leaving 255 prokaryotic vOTUs. Annotation of vOTUs was done using Pharokka, then Phold (74, 75).

The sequencing saturation curve was generated by using seqtk (v1.5) to randomly subsample 5%, 10%, 20%, 40%, 60%, and 80% of reads (76). Read subsamples were then mapped to a FASTA file containing all 255 identified vOTUs using minimap2 (v2.30), then sorted using SAMtools (v2.0.8) (77, 78). CoverM (v0.7.0) was used to determine the breadth of coverage of each vOTU for each subsample (79). The previously established and benchmarked coverage breadth cutoff of ≥75% was used to define “detection” of a given vOTU (33).

The Oral Phage Database was downloaded from the CNGB Sequence Archive of China National GeneBank DataBase (accession number CNP0003685) (16). The Oral Virus Database was downloaded from the OVD GitHub repository (https://github.com/RChGO/OVD/) (35). The Gut Phage Database was downloaded from the GPD repository (https://ftp.ebi.ac.uk/pub/databases/metagenomics/genome_sets/gut_phage_database/) (36). The Gut Virome Database was downloaded from iVirus (https://datacommons.cyverse.org/browse/iplant/home/shared/iVirus/Gregory_and_Zablocki_GVD_Jul2020) (37). Bacteriophage genomes from the NCBI Virus Database were downloaded from NCBI Virus (https://www.ncbi.nlm.nih.gov/labs/virus/vssi/) (38). The Metagenomic Gut Virus Database was downloaded from the repository at https://portal.nersc.gov/MGV/ (39). Sourmash (v4.9.4) was used to sketch the vOTUs and databases with a scaling factor of 1,000 and a k-mer size of 31 (34). Then, Sourmash gather was used to compare the vOTUs to each database with a threshold value of 1,000 bp. Minimap2 (v2.17) was used to create a .mmi file from the combined databases and align the vOTUs to database genomes individually (77).

The DefenseFinder web instance (v2.0.0) was used to identify antiphage defense-related genes within the vOTUs (40). Seaborn Python module (v0.13.2) was used to generate a presence/absence matrix, and Jaccard distance was used to cluster the genes and vOTUs (41, 42).

## Data Availability

Data for this project are available on BioProject (PRJNA1468665) with BioSample accession number SAMN60297583. All reads generated are available in the Sequence Read Archive (SRR38838431). All assembled and annotated vOTUs are available in GenBank (PZ482476–PZ482730).

## SUPPLEMENTAL MATERIAL

Fig. S1 spectrum.01820-26-s0001.docx Transmission electron micrograph of viral particle preparations from dental wastewater. The image shows an abundance of filamentous phage-like particles.

Table S1 spectrum.01820-26-s0002.xlsx Predicted vOTUs from dental wastewater metagenomic sequencing.

ASM does not own the copyrights to Supplemental Material that may be linked to, or accessed through, an article. The authors have granted ASM a non-exclusive, world-wide license to publish the Supplemental Material files. Please contact the corresponding author directly for reuse.

## References

[1] Brives C, Pourraz J. 2020. Phage therapy as a potential solution in the fight against AMR: obstacles and possible futures. Palgrave Commun 6:100. doi:10.1057/s41599-020-0478-4

[2] Cui L, Watanabe S, Miyanaga K, Kiga K, Sasahara T, Aiba Y, Tan X-E, Veeranarayanan S, Thitiananpakorn K, Nguyen HM, Wannigama DL. 2024. A comprehensive review on phage therapy and phage-based drug development. Antibiotics (Basel) 13:870. doi:10.3390/antibiotics13090870 39335043 PMC11428490

[3] Strathdee SA, Hatfull GF, Mutalik VK, Schooley RT. 2023. Phage therapy: From biological mechanisms to future directions. Cell 186:17–31. doi:10.1016/j.cell.2022.11.017 36608652 PMC9827498

[4] Skurnik M, Alkalay-Oren S, Boon M, Clokie M, Sicheritz-Pontén T, Dąbrowska K, Hatfull GF, Hazan R, Jalasvuori M, Kiljunen S, Lavigne R, Malik DJ, Nir-Paz R, Pirnay J-P. 2025. Phage therapy. Nat Rev Methods Primers 5:9. doi:10.1038/s43586-024-00377-5

[5] Hyman P. 2019. Phages for phage therapy: isolation, characterization, and host range breadth. Pharmaceuticals 12:35. doi:10.3390/ph12010035 30862020 PMC6469166

[6] Leprince A, Somerville V, Addablah AA, Morency C, Moineau S. 2026. Phage host range: determinants, dynamics and applications. Nat Rev Microbiol 24:518–538. doi:10.1038/s41579-026-01306-x 42026225

[7] Stewart EJ. 2012. Growing unculturable bacteria. J Bacteriol 194:4151–4160. doi:10.1128/JB.00345-12 22661685 PMC3416243

[8] Inglis LK, Edwards RA. 2022. How metagenomics has transformed our understanding of bacteriophages in microbiome research. Microorganisms 10:1671. doi:10.3390/microorganisms10081671 36014086 PMC9415785

[9] Howard KC, Gonzalez OA, Garneau-Tsodikova S. 2021. *Porphyromonas gingivalis*: where do we stand in our battle against this oral pathogen? RSC Med Chem 12:666–704. doi:10.1039/d0md00424c 34124669 PMC8152699

[10] Szafrański SP, Winkel A, Stiesch M. 2017. The use of bacteriophages to biocontrol oral biofilms. J Biotechnol 250:29–44. doi:10.1016/j.jbiotec.2017.01.002 28108235

[11] Kabwe M, Tucci J, Darby I, Dashper S. 2025. Oral bacteriophages and their potential as adjunctive treatments for periodontitis: a narrative review. J Oral Microbiol 17:2469890. doi:10.1080/20002297.2025.2469890 40013014 PMC11864011

[12] Sandmeier H, Bär K, Meyer J. 1993. Search for bacteriophages of black-pigmented Gram-negative anaerobes from dental plaque. FEMS Immunology & Medical Microbiology 6:193–194. doi:10.1111/j.1574-695X.1993.tb00324.x 8390892

[13] Szafranksi S, Slots J, Steisch M. 2021. The human oral phageome. Periodontology 2000. doi:10.1111/prd.12363 33690937

[14] Carr VR, Shkoporov A, Gomez-Cabrero D, Mullany P, Hill C, Moyes DL. 2020. The human oral phageome is highly diverse and rich in jumbo phages. bioRxiv. doi:10.1101/2020.07.06.186817

[15] Nayfach S, Camargo AP, Schulz F, Eloe-Fadrosh E, Roux S, Kyrpides NC. 2021. CheckV assesses the quality and completeness of metagenome-assembled viral genomes. Nat Biotechnol 39:578–585. doi:10.1038/s41587-020-00774-7 33349699 PMC8116208

[16] Jie Z, Liang H, Meng Y, Zhang J, Zhang T, Li W, Lin X, Hu T, Han M, Liang W, Ju Y, Tong X, Jin X, Xu X, Zhang W, Wang J, Yang H, Kristiansen K, Xiao L, Zou Y. 2025. Integrating metagenomics and cultivation unveils oral phage diversity and potential impact on hosts. NPJ Biofilms Microbiomes 11:145. doi:10.1038/s41522-025-00773-z 40715125 PMC12297358

[17] Fillol-Salom A, Rostøl JT, Ojiogu AD, Chen J, Douce G, Humphrey S, Penadés JR. 2022. Bacteriophages benefit from mobilizing pathogenicity islands encoding immune systems against competitors. Cell 185:3248–3262. doi:10.1016/j.cell.2022.07.014 35985290

[18] Kuang X, Gorzynski J, Touchon M, Shkoporov A, Rocha EPC, Fitzgerald JR, Chen J, Rostøl JT, Penadés JR. 2026. Bacteriophages mobilize bacterial defense systems via lateral transduction. Sci Adv 12:4. doi:10.1126/sciadv.adx5749 PMC12829572 41576165

[19] Duyvejonck H, Merabishvili M, Vaneechoutte M, de Soir S, Wright R, Friman V-P, Verbeken G, De Vos D, Pirnay J-P, Van Mechelen E, Vermeulen SJT. 2021. Evaluation of the stability of bacteriophages in different solutions suitable for the production of magistral preparations in Belgium. Viruses 13:865. doi:10.3390/v13050865 34066841 PMC8151234

[20] Guo J, Bolduc B, Zayed AA, Varsani A, Dominguez-Huerta G, Delmont TO, Pratama AA, Gazitúa MC, Vik D, Sullivan MB, Roux S. 2021. VirSorter2: a multi-classifier, expert-guided approach to detect diverse DNA and RNA viruses. Microbiome 9:37. doi:10.1186/s40168-020-00990-y 33522966 PMC7852108

[21] Camargo AP, Roux S, Schulz F, Babinski M, Xu Y, Hu B, Chain PSG, Nayfach S, Kyrpides NC. 2024. Identification of mobile genetic elements with geNomad. Nat Biotechnol 42:1303–1312. doi:10.1038/s41587-023-01953-y 37735266 PMC11324519

[22] Shang J, Tang X, Guo R, Sun Y. 2022. Accurate identification of bacteriophages from metagenomic data using transformer. Brief Bioinformatics 23. doi:10.1093/bib/bbac258 PMC9294416 35769000

[23] Kieft K, Zhou Z, Anantharaman K. 2020. VIBRANT: automated recovery, annotation and curation of microbial viruses, and evaluation of viral community function from genomic sequences. Microbiome 8:90. doi:10.1186/s40168-020-00867-0 32522236 PMC7288430

[24] Hegarty B, Riddell V J, Bastien E, Langenfeld K, Lindback M, Saini JS, Wing A, Zhang J, Duhaime M. 2024. Benchmarking informatics approaches for virus discovery: caution is needed when combining *In silico* identification methods . mSystems 9:e01105–23. doi:10.1128/msystems.01105-23 38376167 PMC10949488

[25] Wu L-Y, Wijesekara Y, Piedade GJ, Pappas N, Brussaard CPD, Dutilh BE. 2024. Benchmarking bioinformatic virus identification tools using real-world metagenomic data across biomes. Genome Biol 25:97. doi:10.1186/s13059-024-03236-4 38622738 PMC11020464

[3] Kosmopoulos JC, Anantharaman K. 2025. Viral dark matter: illuminating protein function, ecology, and biotechnological promises. Biochemistry 64:4609–4627. doi:10.1021/acs.biochem.5c00349 41264852 PMC12713726

[27] Roux S, Adriaenssens EM, Dutilh BE, Koonin EV, Kropinski AM, Krupovic M, Kuhn JH, Lavigne R, Brister JR, Varsani A, et al.. 2019. Minimum information about an uncultivated virus genome (MIUViG). Nat Biotechnol 37:29–37. doi:10.1038/nbt.4306 30556814 PMC6871006

[28] Shang Jiayu, Tang X, Sun Y. 2023. PhaTYP: predicting the lifestyle for bacteriophages using BERT. Brief Bioinformatics 24. doi:10.1093/bib/bbac487 PMC9851330 36659812

[29] Shang J, Sun Y. 2022. CHERRY: a computational metHod for accuratE pRediction of virus–pRokarYotic interactions using a graph encoder–decoder model. Brief Bioinformatics 23:bbac182. doi:10.1093/bib/bbac182 35595715 PMC9487644

[30] Abranches J, Zeng L, Kajfasz JK, Palmer SR, Chakraborty B, Wen ZT, Richards VP, Brady LJ, Lemos JA. 2018. Biology of oral *Streptococci*. Microbiol Spectr 6. doi:10.1128/microbiolspec.GPP3-0042-2018 PMC6287261 30338752

[31] Könönen E, Gursoy UK. 2021. Oral *Prevotella* species and their connection to events of clinical relevance in gastrointestinal and respiratory tracts. Front Microbiol 12:798763. doi:10.3389/fmicb.2021.798763 35069501 PMC8770924

[32] Könönen E, Fteita D, Gursoy UK, Gursoy M. 2022. *Prevotella* species as oral residents and infectious agents with potential impact on systemic conditions. J Oral Microbiol 14:2079814. doi:10.1080/20002297.2022.2079814 36393976 PMC9662046

[33] Roux S, Emerson JB, Eloe-Fadrosh EA, Sullivan MB. 2017. Benchmarking viromics: an *In silico* evaluation of metagenome-enabled estimates of viral community composition and diversity. PeerJ 5:e3817. doi:10.7717/peerj.3817 28948103 PMC5610896

[34] Pierce NT, Irber L, Reiter T, Brooks P, Brown CT. 2019. Large-scale sequence comparisons with sourmash. F1000Res 8:1006. doi:10.12688/f1000research.19675.1 31508216 PMC6720031

[35] Li S, Guo R, Zhang Y, Li P, Chen F, Wang X, Li J, Jie Z, Lv Q, Jin H, Wang G, Yan Q. 2022. A catalog of 48,425 nonredundant viruses from oral metagenomes expands the horizon of the human oral virome. iScience 25:104418. doi:10.1016/j.isci.2022.104418 35663034 PMC9160773

[36] Camarillo-Guerrero LF, Almeida A, Rangel-Pineros G, Finn RD, Lawley TD. 2021. Massive expansion of human gut bacteriophage diversity. Cell 184:1098–1109. doi:10.1016/j.cell.2021.01.029 33606979 PMC7895897

[37] Gregory AC, Zablocki O, Zayed AA, Howell A, Bolduc B, Sullivan MB. 2020. The gut virome database reveals age-dependent patterns of virome diversity in the human gut. Cell Host Microbe 28:724–740. doi:10.1016/j.chom.2020.08.003 32841606 PMC7443397

[38] Brister JR, Ako-adjei D, Bao Y, Blinkova O. 2015. NCBI viral genomes resource. Nucleic Acids Res 43:D571–D577. doi:10.1093/nar/gku1207 25428358 PMC4383986

[39] Nayfach S, Páez-Espino D, Call L, Low SJ, Sberro H, Ivanova NN, Proal AD, Fischbach MA, Bhatt AS, Hugenholtz P, Kyrpides NC. 2021. Metagenomic compendium of 189,680 DNA viruses from the human gut microbiome. Nat Microbiol 6:960–970. doi:10.1038/s41564-021-00928-6 34168315 PMC8241571

[40] Tesson F, Hervé A, Mordret E, Touchon M, d’Humières C, Cury J, Bernheim A. 2022. Systematic and quantitative view of the antiviral arsenal of prokaryotes. Nat Commun 13:2561. doi:10.1038/s41467-022-30269-9 35538097 PMC9090908

[41] Hancock JM. 2014. Jaccard distance (jaccard index, jaccard similarity coefficient), dictionary of bioinformatics and computational biology. John Wiley & Sons, Ltd.

[42] Waskom ML. 2021. Seaborn: statistical data visualization. JOSS 6:3021. doi:10.21105/joss.03021

[43] Liang Q, Richey ST, Ur SN, Ye Q, Lau RK, Corbett KD. 2022. Structure and activity of a bacterial defense-associated 3’-5’ exonuclease. Protein Sci 31:e4374. doi:10.1002/pro.4374 35762727 PMC9214754

[44] LeRoux M, Srikant S, Teodoro GIC, Zhang T, Littlehale ML, Doron S, Badiee M, Leung AKL, Sorek R, Laub MT. 2022. The DarTG toxin-antitoxin system provides phage defence by ADP-ribosylating viral DNA. Nat Microbiol 7:1028–1040. doi:10.1038/s41564-022-01153-5 35725776 PMC9250638

[45] DeWeirdt PC, Mahoney EM, Laub MT. 2026. Defensepredictor: a machine learning model to discover prokaryotic immune systems. Science 392:eadv7924. doi:10.1126/science.adv7924 41926577 PMC13092281

[46] Pacheco-Acosta S, Castro-Toro G, Rojas-Villalobos C, Valenzuela C, Haristoy JJ, Zapata-Araya A, Moya-Beltrán A, Sepúlveda-Rebolledo P, Pérez-Rueda E, Ulloa R, Giaveno A, Issotta F, Díez B, Beard S, Quatrini R. 2025. Exploring the eco-evolutionary role of plasmids and defense systems in “*Fervidacidithiobacillus caldus*” extreme acidophile. Front Microbiol 16:1610279. doi:10.3389/fmicb.2025.1610279 40862153 PMC12375680

[47] Ishikawa J, Yoneyama K, Azam AH, Nagao A, Mitsuda Y, Nakazaki R, Chihara K, Hiraizumi M, Yamashita K, Suzuki T, Kiga K, Nishimasu H. 2025. Structural mechanism of the retron-Eco7 anti-phage defense system. Nat Commun 16:10821. doi:10.1038/s41467-025-66589-9 41330933 PMC12673091

[48] Wang B, Hoffman RD, Hou Y-M, Li H. 2026. Structural basis for retron co-option of anti-phage ATPase-nuclease. Nat Struct Mol Biol 33:53–62. doi:10.1038/s41594-025-01702-6 41174277 PMC12819146

[49] Franco Álvarez M, Jardi Cuadrado A, Fernández Cambeiro MF, Domínguez Lago A, Díaz Peromingo JA. 2025. *Veillonella atypica* bacteraemia: case report and literature review. IDCases 40:e02194. doi:10.1016/j.idcr.2025.e02194 40129760 PMC11930363

[50] Hiroki H, Shiiki J, Handa A, Totsuka M, Nakamura O. 1976. Isolation of bacteriophages specific for the genus *Veillonella*. Arch Oral Biol 21:215–217. doi:10.1016/0003-9969(76)90132-1 1065266

[51] McGinnis S, Madden TL. 2004. BLAST: at the core of a powerful and diverse set of sequence analysis tools. Nucleic Acids Res 32:W20–W25. doi:10.1093/nar/gkh435 15215342 PMC441573

[52] Moradi J, Berggreen E, Bunæs DF, Bolstad AI, Bertelsen RJ. 2025. Microbiome composition and metabolic pathways in shallow and deep periodontal pockets. Sci Rep 15:12926. doi:10.1038/s41598-025-97531-0 40234709 PMC12000285

[53] Chukkapalli SS, Rivera-Kweh MF, Velsko IM, Chen H, Zheng D, Bhattacharyya I, Gangula PR, Lucas AR, Kesavalu L. 2015. Chronic oral infection with major periodontal bacteria *Tannerella forsythia* modulates systemic atherosclerosis risk factors and inflammatory markers. Pathog Dis 73:ftv009. doi:10.1093/femspd/ftv009 25663343 PMC4542842

[54] Cheeseman SL, Hiom SJ, Weightman AJ, Wade WG. 1996. Phylogeny of oral asaccharolytic *Eubacterium* species determined by 16S Ribosomal dna sequence comparison and proposal of *Eubacterium infirmum* sp. nov. and *Eubacterium tardum* sp. nov. Int J Syst Bacteriol 46:957–959. doi:10.1099/00207713-46-4-957 8863423

[55] Matrishin CB, Haase EM, Dewhirst FE, Mark Welch JL, Miranda-Sanchez F, Chen T, MacFarland DC, Kauffman KM. 2023. Phages are unrecognized players in the ecology of the oral pathogen *Porphyromonas gingivalis*. Microbiome 11:161. doi:10.1186/s40168-023-01607-w 37491415 PMC10367356

[56] Russell DA, Hatfull GF. 2017. PhagesDB: the actinobacteriophage database. Bioinformatics 33:784–786. doi:10.1093/bioinformatics/btw711 28365761 PMC5860397

[57] Cardillo M, Barbera G, Sanfilippo M, Comparato C. 2025. *Rothia dentocariosa* and catheter–related bloodstream infection: rare involvement of a typically non–pathogenic bacterium in a pediatric case of pompe disease. what is the appropriate treatment? European Heart J Suppl 27. doi:10.1093/eurheartjsupp/suaf076.165

[58] Takeuchi Y, Umeda M, Sakamoto M, Benno Y, Huang Y, Ishikawa I. 2001. *Treponema socranskii, Treponema denticola*, and *Porphyromonas gingivalis* are associated with severity of periodontal tissue destruction. J Periodontol 72:1354–1363. doi:10.1902/jop.2001.72.10.1354 11699477

[59] Shrimali T, Malhotra S, Relhan N, Tak V, Choudhary SK, Gupta N, Singh AK. 2023. *Streptococcus parasanguinis*: an emerging pathogen causing neonatal endocarditis: a case report. Access Microbiol 5:000576. doi:10.1099/acmi.0.000576.v4 PMC10323779 37424549

[60] Geng J, Chiu C-H, Tang P, Chen Y, Shieh H-R, Hu S, Chen Y-YM. 2012. Complete genome and transcriptomes of *Streptococcus parasanguinis* FW213: phylogenic relations and potential virulence mechanisms. PLoS One 7:e34769. doi:10.1371/journal.pone.0034769 22529932 PMC3329508

[61] Cross KL, Chirania P, Xiong W, Beall CJ, Elkins JG, Giannone RJ, Griffen AL, Guss AM, Hettich RL, Joshi SS, Mokrzan EM, Martin RK, Zhulin IB, Leys EJ, Podar M. 2018. Insights into the evolution of host association through the isolation and characterization of a novel human periodontal pathobiont, *Desulfobulbus oralis*. mBio 9. doi:10.1128/mBio.02061-17 PMC5850319 29535201

[62] Yao F, He J, Nyaruaba R, Wei H, Li Y. 2025. Unveiling the role of phages in shaping the periodontal microbial ecosystem. mSystems 10:e00201–25. doi:10.1128/msystems.00201-25 40152610 PMC12013270

[63] Rakonjac J, Das B, Derda R. 2016. Editorial: filamentous bacteriophage in bio/nano/technology, bacterial pathogenesis and ecology. Front Microbiol 7. doi:10.3389/fmicb.2016.02109 PMC5179506 28066406

[64] Rakonjac J. 2022. Filamentous Bacteriophages: Biology and Applications, p 1–15. eLS. John Wiley & Sons, Ltd.

[65] Kubota N, Scribner MR, Cooper VS. 2025. Filamentous cheater phages drive bacterial and phage populations to lower fitness. Curr Biol 35:5220–5229. doi:10.1016/j.cub.2025.09.029 41043417

[66] Rice SA, Tan CH, Mikkelsen PJ, Kung V, Woo J, Tay M, Hauser A, McDougald D, Webb JS, Kjelleberg S. 2009. The biofilm life cycle and virulence of *Pseudomonas aeruginosa* are dependent on a filamentous prophage. ISME J 3:271–282. doi:10.1038/ismej.2008.109 19005496 PMC2648530

[67] Whiteley M, Bangera MG, Bumgarner RE, Parsek MR, Teitzel GM, Lory S, Greenberg EP. 2001. Gene expression in *Pseudomonas aeruginosa* biofilms. Nature 413:860–864. doi:10.1038/35101627 11677611

[68] Pant A, Das B, Bhadra RK. 2020. CTX phage of Vibrio cholerae: genomics and applications. Vaccine 38 Suppl 1:A7–A12. doi:10.1016/j.vaccine.2019.06.034 31272871

[69] Kolmogorov M, Yuan J, Lin Y, Pevzner PA. 2019. Assembly of long, error-prone reads using repeat graphs. Nat Biotechnol 37:540–546. doi:10.1038/s41587-019-0072-8 30936562

[70] Abueg LAL, Afgan E, Allart O, Awan AH, Bacon WA, Baker D, Bassetti M, Batut B, Bernt M, Blankenberg D, et al.. 2024. The galaxy platform for accessible, reproducible, and collaborative data analyses: 2024 update. Nucleic Acids Res 52:W83–W94. doi:10.1093/nar/gkae410 38769056 PMC11223835

[71] Steinegger M, Söding J. 2017. MMseqs2: sensitive protein sequence searching for the analysis of massive data sets. Bioinformatics. doi:10.1101/079681 29035372

[72] Shang J, Jiang J, Sun Y. 2021. Bacteriophage classification for assembled contigs using graph convolutional network. Bioinformatics 37:i25–i33. doi:10.1093/bioinformatics/btab293 34252923 PMC8275337

[73] Shang J, Peng C, Liao H, Tang X, Sun Y. 2023. PhaBOX: a web server for identifying and characterizing phage contigs in metagenomic data. Bioinformatics Advances 3:vbad101. doi:10.1093/bioadv/vbad101 37641717 PMC10460485

[74] Bouras G, Nepal R, Houtak G, Psaltis AJ, Wormald P-J, Vreugde S. 2023. Pharokka: a fast scalable bacteriophage annotation tool. Bioinformatics 39:btac776. doi:10.1093/bioinformatics/btac776 36453861 PMC9805569

[75] Bouras George, Grigson SR, Mirdita M, Heinzinger M, Papudeshi B, Mallawaarachchi V, Green R, Kim RS, Mihalia V, Psaltis AJ, Wormald P-J, Vreugde S, Steinegger M, Edwards RA. 2026. Protein structure-informed bacteriophage genome annotation with Phold. Nucleic Acids Res 54. doi:10.1093/nar/gkaf1448 PMC12774648 41495893

[76] Li H. 2025. Seqtk. Available from: https://github.com/lh3/seqtk/?tab=MIT-1-ov-file

[77] Li H 2018. Minimap2: pairwise alignment for nucleotide sequences. Bioinformatics 34:3094–3100. doi:10.1093/bioinformatics/bty191 29750242 PMC6137996

[78] Li Heng, Handsaker B, Wysoker A, Fennell T, Ruan J, Homer N, Marth G, Abecasis G, Durbin R, 1000 Genome Project Data Processing Subgroup. 2009. The sequence alignment/map format and SAMtools. Bioinformatics 25:2078–2079. doi:10.1093/bioinformatics/btp352 19505943 PMC2723002

[79] Aroney STN, Newell RJP, Nissen JN, Camargo AP, Tyson GW, Woodcroft BJ. 2025. CoverM: read alignment statistics for metagenomics. Bioinformatics 41. doi:10.1093/bioinformatics/btaf147 PMC11993303 40193404

