# Supplementary material for: Dental wastewater reveals a hidden reservoir of oral bacteriophage diversity
Source: Microbiol Spectr. 2026 Aug 12;14(9):e01820-26. doi: 10.1128/spectrum.01820-26 (PMC13532340; doi:10.1128/spectrum.01820-26)
Supplement: Supplementary file 1 [file spectrum.01820-26-s0001.docx]

**Fig. S1.** Transmission electron micrograph of viral particle preparations from dental wastewater. Image shows abundance of filamentous phage-like particles. Filamentous phage-like particles were observed in multiple fields of view and varied substantially in length. Black scale bar in the upper right corner represents 100 nm.
